# “If people are hesitant at all, you just want a really big front door”: a rapid qualitative interview study on the Luton COVID-19 vaccination outreach clinics

**DOI:** 10.1186/s12889-023-15016-z

**Published:** 2023-03-07

**Authors:** M. Logue, C. Haylock, C. Scarborough, J. Mackenzie

**Affiliations:** 1 Hertfordshire County Council, Public Health Evidence and Intelligence, Public Health, County Hall, Pegs Lane, Hertford, SG13 8DQ UK; 2 University of Southampton, Primary Care, Medical Education and Population Science, Aldermoor Health Centre, Southampton, SO16 5ST UK; 3 Luton Borough Council Public Health, Third floor, Arndale House, The Mall, Luton, LU1 2LJ UK

**Keywords:** COVID-19, Vaccination, Outreach clinics, Qualitative, Pandemic, Community

## Abstract

**Background:**

There is a lack of evidence on the usefulness, practicality, and acceptance of vaccination outreach clinics in the community especially during pandemics. In this qualitative study, we explored the experiences, motivations and perceptions of service users, health professionals, strategic staff, volunteers, and community workers involved in the COVID-19 vaccination outreach clinics in Luton.

**Methods:**

Semi structured face to face, telephone, online interviews, and focus groups were conducted with 31 participants including health professionals, strategic staff, volunteers, community workers and service users. The Framework Method was used to analyse the data and generate themes.

**Results:**

Service users expressed positivity towards the convenience and familiarity of the location of the vaccination outreach clinics and the flexibility of receiving the vaccination in a local setting. Participants involved in the planning and delivery of the service commented on the worthwhile and rewarding experience but suggested more attention should be given to preparation time, service user recruitment, the working environment, and staff welfare.

**Conclusions:**

The COVID-19 mobile vaccination outreach clinics in Luton tested and developed a different model of service delivery and demonstrated a collaborative way of working: “taking the health service to the patient, not the patient to the health service”. Planning and local community engagement were seen as key to successful delivery of a mobile healthcare service.

## Introduction

The use of vaccination outreach clinics during the COVID-19 pandemic were a common occurrence throughout the UK and the USA especially in areas where uptake of the vaccine was low [1, 2]. A recent article by Velasquez, Nolen and Martin [3] suggested that vaccination outreach clinics “work”, and the framework should be rolled out more widely to communities to achieve health equity.

During Spring 2021, Luton had a lower uptake of the vaccination and had higher levels of COVID-19 infection compared to other areas of the UK [4]. Luton is a unitary authority situated in the southeast of England with a population of 225, 261 [5]. According to the 2021 census, 45% of the population is White; 37% Asian or Asian British; 10% Black or Black British and 4% is of Mixed ethnicity. There is a significant population of South Asian descent, mainly Pakistani (18%), Bangladeshi (9%) and Indian (5%). When compared nationally, Luton has a younger resident population; the median age of Luton residents is 34 compared to 40 in England and Wales [6]. 51 of the 121 (42%) Lower Super Output Areas (LSOAs; Office for National Statistics administrative areas) in Luton are in the top 30% deprived areas nationally [7].

To address the low uptake of vaccinations and high COVID infection rates, Luton Borough Council Public Health and the local CCGs worked together with the Luton community to rapidly develop a locally tailored approach by “intensifying existing or novel local engagement activity” [8].

Outreach vaccination clinics were deployed in the Luton area in March (26-28th) and subsequently in April (23-25th) 2021 to deliver vaccinations to the local community. Six locations around Luton were chosen to deliver the vaccinations. These locations were:

- Sainsbury’s, Bury Park
- Town Hall, in Luton town centre,
- Central Mosque, Bury Park,
- Redgrave vaccination site (walk-in lane)
- Inspire vaccination site (walk in lane)
- Luton Irish Forum.

In choosing these locations, stakeholders used local knowledge and intelligence of the resident population to assess the suitability of sites. A bus was acquired from Luton Airport and re-purposed for the mobile clinic situated at the Sainsbury’s, Bury Park car park site. This allowed for flexibility of location and convenience for service users.

Currently there is sparse published information in the literature on the implementation of vaccination outreach clinics in the UK. The aim of the study was to explore the experiences, motivations and perceptions of service users, health professionals, volunteers, strategic staff, and community workers involved in the COVID-19 vaccination outreach clinics in Luton.

## Methods

Semi-structured qualitative interviews and focus groups were conducted with service users, volunteers, health professionals, strategic/planning groups, and community workers from April 23rd to May 3rd, 2021, during the COVID-19 pandemic (See Table 1). Service users and onsite staff were recruited opportunistically for interviews from the vaccination outreach clinic sites (face-to-face; *n* = 17). The remaining participants were interviewed by telephone (*n* = 6) or online through Microsoft Teams (*n* = 8). For the face-to-face interviews people wore facemasks and stood two metres apart.

**Table 1:** Characteristics of interview participants

***Participant Type and Gender***	***Number of participants***
Service Users (Including community workers)	**15**
*Male*	*10*
*Female*	*5*
Health Professionals	**8**
*Male*	*2*
*Female*	*6*
Volunteers	**4**
*Male*	*2*
*Female*	*2*
Strategic Group	**4**
*Male*	*3*
*Female*	*1*
**Total**	**31**

The interview guide was developed from the aims of the study and with discussions with the Luton Borough Council Public Health team. Findings from earlier interviews were used iteratively to inform subsequent data collection and questions were refocused and adopted to evolving themes. Data collection continued until no more important novel responses were received.

Prior to each interview/focus group the researcher provided the participant with verbal information regarding the project and reasons why the data was important, its purpose and how it would be stored. The interviews lasted on average 20 minutes for each participant. Focus groups lasted on average an hour for each session. The interviews were digitally recorded (Olympus DS-2500), transcribed in full by a professional transcriber and anonymised with only the researchers knowing the identity of each participant. The transcripts were compared with the original recorded audio file and were amended as required. NVivo v.13® was used to manage the data corpus.

The analytic approach in this study was Framework analysis. Framework analysis is commonly used in rapid research situations [9, 10]. All transcripts were coded on a line-by-line basis by two researchers. The data was saved in an Excel file and the data in the framework was used to create a list of codes and eventually overarching themes.

## Results

Thirty-two people were approached of whom thirty-one participated in an interview or focus group. The themes were developed from analysis of the data corpus. There was a significant amount of rich data in this project therefore the focus was on those themes relevant to the aim of the study.

Three overarching themes were identified in this study:

1. Planning and communication
2. Accessibility and community involvement
3. Vaccination experiences and opinions

### Planning and communication

Much of the initial discussion was around the decisions that were faced by the parties involved in choosing the vaccination sites and organising/supplying the resources needed to deliver the clinics in a short time scale. The challenge of engaging certain communities in the Luton area was frequently mentioned in the interviews.

Even though lack of time was cited as a barrier to successful delivery of the mobile vaccination clinics, there was a positive atmosphere from among those involved in planning and delivery of the clinics (Table 2, Quote 1). Some health professionals suggested decision making around the criteria for eligibility needed to be more flexible when you want maximum recruitment to the outreach clinics (2).

**Table 2:** Planning and communication quotes

***Quote number***	***Supporting quote***	***Participant***
Quote 1	*“Even though it was a rush, the actual operation on the days was very good from the point of view that we very quickly got into a rhythm of understanding what everybody did at each site, what their roles were and how we supported each other through that process. It was very good”*	Manager, male
Quote 2	*“I think if you really want to max out the capacity you’ve got, you almost need to be slightly ahead of the game on the criteria you’re using”*	Health professional, female
Quote 3	*“It has been quite heavily publicised on the local Facebook group, community action groups, and a few text messages going around, sort of, “Right, get yourself down there, there is no queue,” so that’s why I am here now”*	Service user, male
Quote 4	“*That is because somebody has gone back and said it was really easy to their next-door neighbour. You wouldn’t have got those people if you hadn’t had the access for that day at that site with them not having to book”*	Health professional, female
Quote 5	*“I think, because it’s at short notice, the challenge is to make sure that people know about it. And because the comms wasn’t agreed till I think, I’d say Wednesday evening, that put a very tight timescale on how we could get the word out”*	Community worker, female
Quote 6	*“I thought it was quite good, initially but I think there’s a lot of confusion around who’s allowed to get it done and who isn’t. I think a lot of it’s to do with … I think there’s mixed messages”*	Service user, female
Quote 7	*“The inference was that we would vaccinate all teachers. Then somewhere along the line somebody on social media started putting everybody over 18. Social media wasn’t particularly our friend in that respect”*	Health professional, female

The influence of social media on the dissemination of vaccination information was talked about by every group (3). There was frequent discussion on how community behaviour was influenced by word of mouth (through family and friends). This was an important feature of the vaccination outreach project (3,4). Communicating the correct details about the vaccination outreach clinics to the local community in such a short amount of time was a challenge (5).

Some of the local community were confused around who was eligible for the vaccination and felt the communication was unclear (6). This was compounded by the influence of social media which had a negative impact on the first phase of the project and led to miscommunication around the inclusion criteria (7).

### Accessibility and community involvement

This theme explored how making the COVID-19 vaccine more accessible to the local community in Luton was perceived by the service users, community workers, health professionals and the strategic group. Many service users commented on the benefit of having the clinics in an easy to reach location (Table 3, Quote 8). Others talked about how mobile or local clinics increased the uptake of the vaccination especially for those people who face obstacles (9). Some mentioned how having the clinics in a familiar place such as a local landmark or a supermarket made people more likely to have the vaccination (10). Having an alternative to receiving the vaccination at a local surgery was seen as convenient and was considered useful in removing barriers (11).

**Table 3:** Accessibility and community involvement quotes

***Quote number***	***Supporting quote***	***Participant***
Quote 8	“*It’s a wonderful idea, yes. It makes the vaccine far more accessible”*	Service user, male
Quote 9	*“Superb idea, just brings it closer to the people who maybe don’t live as close to some of the main vaccine centres as others, or perhaps don’t have the transport to get there”*	Community worker, male
Quote 10	*“I was surprised at how many people came just because they’d been to Sainsbury’s to do their shopping”*	Health professional, female
Quote 11	*“I think the idea of them, more localised, is good and not necessarily tied to your surgery is fine, as well because that’s also another blocker, I would say, as far as that goes “*	Community worker, male
Quote 12	*“And I suppose if people are on the fence about it, it might push them over into choosing to come for it, yeah”*	Service user, male
Quote 13	*“I vaccinated an elderly lady and her daughter on the bench next to the bus. We went to people’s car if their loved one couldn’t get out the car”*	Health professional, female
Quote 14	*“The Sainsbury’s one wasn’t even that busy. I felt like maybe, they refused people a lot more. Whereas with the other one, they were quite welcoming and wanting people to come. I think they were a little bit more encouraging. Whereas that, it was like a military fort and how dare you?”* (Laughter)	Service user, female
Quote 15	*“There’s something about getting buy-in from the community rather than doing it to them saying to them, “How can you help us get bums on seats or jabs in arms?””*	Strategic group, male
Quote 16	*“What we were finding is that someone else in the queue they knew was then willing to translate for them. Again, if you’re in vaccination centre you wouldn’t have had that necessarily with that help and that guidance, which was absolutely great”.*	Health professional, female
Quote 17	*“You know, normally we’re telling people, “Don’t smoke, don’t drink, don’t have a doughnut,” you know, kind of thing, and so here we were with something actually people wanted to hear and wanted to see, and so just to be involved in that was really, really special”*	Strategic group, male

According to one service user, bringing vaccines to the community would encourage people who were undecided to get the vaccination (12). Health professionals described the benefits of the location and the flexibility of working from a bus (13). Another service user described her experiences of trying to get the vaccination from two different sites (14).

The topic of community appeared regularly throughout the interviews. The idea of working together was seen as positive especially amid the COVID-19 pandemic and was essential for the successful delivery of the project. It was evident through the interviews that the project was challenging but involved a great deal of teamwork and benevolence. Working together with the community rather than giving orders was seen as the way forward to delivering vaccinations. Some saw the mobile vaccination model in the community as a way of removing barriers (15).

Difficulties with language and translation were overcome with goodwill in the community (16). There was positive feedback from the public on the vaccination outreach clinics which is not always the case in public health according to some public health professionals (17).

### Vaccination experiences and opinions

There were a wide range of opinions on how the outreach clinics were operationalised. Most service users were impressed with how the vaccination was administered and with the professionalism and efficiency of the staff working at the different sites (Table 4, Quote 18).

**Table 4:** Vaccination experiences and opinions

***Quote number***	***Supporting quote***	***Participant***
Quote 18	*“The people that were running them were very professional and getting things done very quickly and very swiftly”*	Service user, female
Quote 19	*“When I had my first jab, I found it so emotional because it’s the next step to opening up, it’s the new normal coming, you know”*	Service user, female
Quote 20	*“Me personally, I’ve taken the jab myself and, in my experience, its saved my life, to tell you the truth but if you didn’t, you’ve got problems. If you don’t get it, you are like a loose cannon”*	Community representative, male
Quote 21	*“I think some of the side effects hasn’t helped in recent times, and I think that kind of spreads a rumour mill. I know I was aware of one person that turned up today and, when they found it was AstraZeneca, they went away”*	Volunteer, female
Quote 22	*“And if people are hesitant at all, you just want a really big front door, and it makes the door … the front door very big that way. Because the booking is quite narrow really, and it’s fine if you fit. But, I guess, if you’re nervous or you’ve got questions, or you’re busy, or any of those things, it can be really difficult”*	Volunteer, female
Quote 23	*“It made me think about whether it was a safe place for them to not be okay. If they fainted or something at the mosque it was like they were amongst friends, so they knew they would be well cared for. I didn’t know whether that was one of the things that would have stopped them from going to a big mass vaccine centre where they didn’t know people”*	Health professional, female
Quote 24	*“I think it was that whole buzz of a different project and a different way of doing it and how would it go. Obviously, there’s a bit of anxiety there because you haven’t got the same support around you either. You do worry”*	Health professional, female
Quote 25	*“It felt like giving everybody a gift because it was a bonus to what … nobody had great expectations around it, but it did feel like every vaccine we gave was a gift. It was like being Santa. People were so grateful”*	Health professional, female
Quote 26	*“I think we need to be a bit more flexible about the hours that we do. Don’t do 10:00 to 4:00 but maybe on the Friday we do 12:00 to 7:00 as we did in the previous one for the static sites”*	Health professional, female
Quote 27	*“I think because we have managed the buses successfully there’s perhaps starting to be a bit of perception from other parts of the country that this is a pick up a bus, drop and go, and it can be underestimated the amount of planning and operations that are required”*	Health professional, female
Quote 28	*“Every one person that you capture that is really vulnerable makes such a difference. It’s really powerful story, so it is understanding all those stories”*	Health professional, female
Quote 29	*“So, I actually think taking the health service to the patient, not the patient to the health service, is the way forward and we’ve got to think about that”*	Strategic group, male
Quote 30	*“We’ve got to cut through – excuse my language – the red tape and the bureaucracy and I think we must not go back to the old world because otherwise, we’re letting our patients down, our population down, that’s what I would say, as a key challenge”*	Strategic group, male
Quote 31	*“So, I think there’s a challenge there for us about who really are the community leaders, who are the people the community does actually respond to, and it might not be the ones we historically thought they were”*	Strategic group, male
Quote 32	*“It really is about getting that communication right and I think it’s more about boots on the ground, you know, it’s kissing the babies, as you would in the old days, but I think that’s what it’s about, really going out and engaging”*	Strategic group, female

There was a sense that having the vaccination was part of a larger movement to open up society from lockdown (19). A member of one of the communities involved in the project had strong feelings about getting the vaccination and considered her role in the pandemic (20).

There was discussion on why some people were hesitant about receiving the vaccination (21). An important point was made around making the eligibility criteria as flexible as possible as this would reduce vaccination hesitancy and increase uptake (22). A safe and familiar place to have the vaccination was seen as essential (23). Health professionals talked about the new experience of working in mobile vaccination clinics and voiced their concerns about working in an unfamiliar environment (24). Others compared administering the vaccination akin to "Santa" giving presents (25).

The limited opening times of the vaccination outreach clinics was seen as a barrier to the recruitment of service users (26). The complexities of working on a bus should not be underestimated according to one health professional (27). Vaccinating vulnerable populations was seen as a priority but also played a role in the wider impact of outreach clinics (28). Major learning outcomes from this project were around making healthcare more person and community centred and reducing “red tape” (29,30).

The difficulty of identifying and engaging with community leaders was mentioned as a challenge for some communities and more work is needed in this area (31). Communication with the local population at a grassroots level is key to engagement according to some participants (32).

## Discussion

### Main findings of this study

This qualitative study explored the experiences, motivations and perceptions of participants involved in the Luton COVID-19 vaccination outreach clinics. Many service users visited the mobile clinics as they were accessible and in familiar locations such as the Sainsbury’s car park in Bury Park, the Luton Irish Forum, and the Central Mosque. Service users commented that it was more convenient to visit the vaccination outreach clinics than to use the National Booking Service or visit their allocated vaccination centre. Leibowitz et al. [11] found that outreach vaccination clinics were valuable for service users who were concerned about COVID-19 transmission or faced barriers to in-person care. They concluded that mobile health clinics are an underused and valuable method for delivering healthcare for difficult to reach and underserved populations.

Word of mouth and the use of social media were important communication methods regarding the mobile vaccination clinics in the community however these methods also fostered confusing messaging around eligibility. Working with the community and providing a flexible approach to the delivery of the vaccine helped to overcome obstacles (such as lack of transport and language barriers) which some faced in getting the vaccination. Feedback from health professionals suggested the operational hours of the clinics be amended to be more inclusive and appropriate to the needs of the local population.

The vaccination outreach clinics were a success according to feedback from health professionals, volunteers, and other members of the team; a rewarding experience and a worthwhile project to have participated in, however, there were some comments around the working environment, preparation time and staff welfare.

Comments from some participants suggested that by running the vaccination outreach clinics, a different model of service delivery was tested and developed. A new way of working was discussed in terms of “taking the health service to the patient, not the patient to the health service”. Planning and local engagement were seen as key to successful delivery of a mobile healthcare service. According to some participants it is imperative that stakeholders and team members involved in the project have the appropriate skill set, for example, in areas with diverse populations, team members with relevant language skills would be beneficial.

Interestingly, NICE [12] recommended providing multiple opportunities and convenient vaccination locations and times for communities who face barriers to vaccine uptake or may have low vaccine uptake. They suggested that although outreach clinics are associated with additional resource use, this cost is likely to be offset by the benefits of avoiding disease outbreaks and their associated care costs.

### What is already known on this topic

The WHO report [8, 13] on vaccinations suggested that understanding a wide range of factors leads to successful vaccination roll out. The 3Cs framework was created to deal with factors effecting vaccination uptake. This includes confidence, convenience, and complacency. Confidence refers to the safety and effectiveness of vaccines. This trust concerns the system that delivers the vaccines and the competence of health providers. The convenience of vaccination services is of paramount importance when aiming for maximum vaccination uptake. Convenience includes affordability, location, availability, language fluency and health literacy. Complacency occurs when the perception exists that a vaccine is not considered a necessity to prevent disease or illness.

### What the study adds

Our research demonstrates that with a co-production approach it is possible to plan, implement and run vaccination outreach clinics in the community with short notice, however, more work is required to change attitudes and break down structural obstacles to vaccination. This may involve utilising local knowledge to inform design and build trust between healthcare providers and the community around vaccination and delivering clinics in the right location or making them more mobile and accessible, to achieve vaccine equity.

### Limitations of this study

Although every effort was made to recruit a diverse range of participants in this study, it is acknowledged that there may be response bias as the sample interviewed were wholly positive about the mobile vaccination clinics. It would have been useful to interview service users who were refused the vaccination due to not fitting the inclusion criteria or were hesitant about receiving the vaccination.

## Data Availability

The data underlying this article will be shared on reasonable request to the corresponding author.
